# Bioactivity of mycosynthesized nanoparticles assists photothermal therapy of breast cancer cells

**DOI:** 10.1007/s10495-025-02145-6

**Published:** 2025-08-30

**Authors:** Aya Ezzat, Marwa A. Ramadan, Jehane I. Eid, Ola S. Ahmed

**Affiliations:** 1 Zoology Department, Faculty of Science, Cairo University, Giza, Egypt; 2 Department of Laser Application in Metrology, Photochemistry and Agriculture, National Institute of Laser Enhanced Science (NILES), Cairo University (CU), Giza, Egypt; 3 Virology and Immunology Unit, Cancer Biology Department, National Cancer Institute-Cairo University, Cairo, Egypt

**Keywords:** Chaga mushroom, Gold nanoparticles, Mycosynthesized nanoparticles, Photothermal therapy, Breast cancer, LED irradiation

## Abstract

Chaga mushroom (*Inonotus obliquus*) exhibits cytotoxic effects against breast cancer cells. Mycosynthesized nanoparticles, owing to their biodegradability, biocompatibility, and low toxicity, present a promising therapeutic approach. This study explored the cytotoxic potential of gold nanoparticles synthesized using Chaga mushroom extract (AuCh-NPs) combined with Light Emitting Diode (LED) irradiation (530 nm) on human breast cancer (MCF-7) cells, aiming to develop a safe and effective sensitizer for photothermal therapy. The AuCh-NPs were characterized using UV–visible spectroscopy, FTIR, TEM, particle size analysis, and zeta potential measurements. Cytotoxicity was evaluated via MTT assay under LED irradiation with total light exposure 325.8 and 488.7 J cm^−2^, alongside mechanistic studies involving wound healing, autophagy, cell cycle arrest, annexin V analysis, real-time PCR, and comet assays.TEM revealed spherical AuCh-NPs with sizes ranging from 15.4 to 28.9 nm. The MTT assay demonstrated enhanced cytotoxicity under LED irradiation, with AuCh-NPs exhibiting a lower IC50 (5.56 µM) than citrate-capped gold nanoparticles (AuCit-NPs, 7 µM). Cell cycle analysis revealed significant arrest in G0/G1 (91.68%) and S (7.55%) phases, while annexin V analysis confirmed apoptosis induction. Real-time PCR showed upregulation of the pro-apoptotic genes BAX, and the comet assay indicated increased double-strand DNA damage in MCF-7 cells treated with AuCh-NPs compared to AuCit-NPs. These findings highlight the superior selective cytotoxicity of AuCh-NPs against MCF7 cells, positioning them as a promising targeted agent for photothermal therapy in breast cancer treatment.

## Introduction

Breast cancer remains the leading cause of cancer-related mortality among women worldwide, with a five-year survival rate below 50% for patients with metastatic disease [1]. According to the International Agency for Research on Cancer (IIARC), its incidence continues to rise alarmingly despite extensive research efforts to elucidate its complex pathogenesis [2], This heterogeneous malignancy is often driven by dysregulation in hormone receptor genes, including estrogen receptor (ER), progesterone receptor (PR), and human epidermal growth factor receptor 2 (HER2) [2]. Traditional treatments, including surgery, chemotherapy, radiotherapy, and hormone therapy, prolong survival but are limited by severe side effects and systemic toxicity [3, 4].

Recent scientific progress has led to the discovery of alternative approaches based on noble metal nanoparticles, particularly gold and silver nanoparticles (AuNPs and AgNPs), for treating breast cancer [5]. Due to their physiochemical properties, biocompatibility, and multifunctional applications in diagnostics and drug delivery, these nanoparticles have emerged as promising alternatives for targeted breast cancer therapy [6]. Compared to conventional synthesis methods, green synthesis, especially mycosynthesis, offers an eco-friendly, rapid, and high-yield approach to nanoparticle production [7]. However, while noble metal nanoparticles demonstrate potent cytotoxicity and intracellular accumulation in cancer cells, their therapeutic efficacy can be further enhanced through precise functionalization [8].

*Inonotus obliquus* (Chaga mushroom), a medicinal fungus of the *Hymenochaetaceae*family, has been used in traditional medicine since the 16th century for its immunomodulatory, anti-inflammatory, and anticancer properties [9–11]. Its bioactive components, including polyphenols (3,4-dihydroxybenzal acetone), triterpenes (e.g., betulinic acid, inotodiol), and polysaccharides, exert chemopreventive effects by inhibiting tumor proliferation, inducing apoptosis, and stimulating immune responses without harming healthy cells [12–14]. Despite its therapeutic potential, the clinical translation of Chaga extracts has been hindered by the high concentrations required for efficacy [15].

Recent advances in mycosynthesis have enabled the production of biologically active AuNPs using fungal metabolites as reducing and stabilizing agents [16]. Such nanoparticles exhibit selective cytotoxicity toward cancer cells while sparing normal tissues, as demonstrated by *Exiguobacteriumaestuarii*-derived AuNPs, which show antioxidant and anticancer activity against MCF-7 cells [17, 18]. The mycosynthesis of Chaga-mediated gold nanoparticles (AuCh-NPs) leverages the mushroom’s bioactive components to enhance nanoparticle stability and targeting efficiency [17].

Photothermal therapy (PTT) has gained traction as a minimally invasive, highly selective cancer treatment. By combining AuNPs with light irradiation, PTT induces localized hyperthermia, triggering cancer cell death [19, 20]. The photothermal efficiency of AuNPs depends on their size, shape, and surface properties, which can be optimized through eco-friendly synthesis [21]. Thus, in this study, we investigated the synergistic anticancer effects of mycosynthesized AuCh-NPs and citrate-capped AuNPs (AuCit-NPs) under LED irradiation (530 nm, 142 mW, 5 cm distance) against MCF-7 breast cancer cells, evaluating their potential as targeted photothermal agents.

## Materials and methods

This study evaluated the physiochemical properties and anticancer efficiency of Chaga extract, mycosynthesized gold nanoparticles (AuCh-NPs), and citrate-capped gold nanoparticles (AuCit-NPs) combined with photothermal therapy (530 nm LED irradiation) against the human breast cancer cell line MCF-7.

### Preparation of Chaga extract

0.5 g of *Inonotus obliquus* (Chi chaga, Canada) was dissolved in 50 mL deionized water, magnetically stirred (CLE-119, Egypt) for 60 min, and incubated for 24 h at room temperature. The mixture was centrifuged (Benchmark LC-8, China) at 1000 rpm for 10 min to yield 0.005 g/mL aqueous extract [19].

### Mycosynthesis of AuCh-NPs

AuCh-NPs were synthesized by reducing 10 mL of 0.1 mM HAuCl_4_ with 4 mL of Chaga extract using magnetic stirring for 1 h at room temperature. Formation was confirmed by a color change to amber [22].

### Synthesis of AuCit-NPs

AuCit-NPs were chemically synthesized by boiling 5 mL of 0.01 M HAuCl_4_ with 0.44 g sodium citrate and 1 g Polyvinylpyrrolidone (PVP; Sigma-Aldrich) in 30 mL distilled water. The amber color indicated nanoparticle formation [23].

### Characterization of synthesized nanoparticles

#### UV–visible spectrophotometer

Absorption spectra (200–800 nm) were recorded using a Cary-4000 spectrophotometer (PG instrument, UK) with distilled water as a blank [25].

#### Fourier Transform-Infrared Spectroscopy (FTIR)

Functional groups were analyzed using a NICOLET 6700 FTIR spectrometer (Thermo Scientific) with KBr pellets (500–4000 cm^−1^) [26].

#### Transmission Electron Microscope (TEM)

Morphology and size were assessed using a JEOL JEM-1400 (Cairo University Research Park) [25].

#### Zeta potential and Dynamic Light Scattering (DLS)

Surface charge and hydrodynamic diameter were measured using Zetasizer (Malvern Instruments, UK). Hence, the diffusion behavior of molecules in nanoparticle solutions was detected by the Dynamic Light Scattering (DLS) technique. Then, from their scattering pattern, the hydrodynamic radii were calculated based on the diffusion coefficient of Chaga extract, AuCh-NPS, and AuCit-NPs [27].

#### Particle size analysis

Nanoparticle sizes were determined via NanoSight NS500 (MalvernPanalytical) [19].

### Cell culture

MCF-7 cells (National Centre Institute, Egypt) were cultured in RPMI-1640 (10% FBS, 100 U/mL penicillin, 10 µg/mL streptomycin; Gibco) at 37 °C/5% CO_2_. Cells were treated with 2–8 µM of Chaga extract, AuCh-NPs, and AuCit-NPs (four replicates per concentration) [19].

### LED irradiation conditions

Cells were exposed to 530 nm LED (142 mW, 5 cm distance; irradiance: 0.181 W/cm^2^) for 30 min (325.8 J/cm^2^) and 45 min (488.7 J/cm^2^) at room temperature post-incubation [25]. The irradiation spot had a diameter of 1 cm, corresponding to an illuminated area of approximately 0.785 cm^2^.

### MTT assay

Cell viability was evaluated using a standard MTT colorimetric assay. Briefly, MCF-7 Cells were treated with Chaga extract, AuCh-NPs, or AuCit-NPs at concentrations of 2, 4, 6, and 8 µM for 24 h. Following treatment, 0.5 mg/mL MTT reagent (3-(4,5-dimethylthiazol-2-yl)-2,5-diphenyl-2H-tetrazolium bromide; Sigma-Aldrich) was added to each well, and plates were incubated at 37 °C for 4 h in a 5% CO_2_ incubator (Thermo Scientific, USA) to allow viable cells to reduce MTT to formazan crystals. The reaction was terminated by adding 100 µL of solubilization solution (Sigma-Aldrich) to dissolve the formazan crystals [15]. Absorbance was measured at 570 nm using a SpectraMax M5 microplate reader (Molecular Devices, USA).

Cell viability was calculated as:

$$ {\text{Cell viability }}\left( {{1}00\% } \right) = \left( {{\text{ODS}}/{\text{ODC}}} \right) \times {1}00 $$

where ODS represents the mean optical density of treated samples, and ODC the mean optical density of untreated controls. Dose–response curves and IC50 values were calculated using nonlinear regression analysis in GraphPad Prism 8.0.2 software February 06, 2019 (GraphPad Software, USA).

### Wound healing assay

Confluent monolayers of MCF-7 cells from all groups were scratched with a sterile 200 µL pipette tip (Thermo Scientific, USA). The wells were then washed with PBS (Gibco, USA) to remove cellular debris and incubated for 24 h in an incubator (Thermo Scientific, USA). After incubation, cell migration was visualized in all directions (top, middle, and bottom) using an inverted microscope (Thermo Scientific, USA) at 20 × magnification, and digital images were captured. The migration distance between scratch edges was quantified in pixels using ImageJ (S.A., France) version 1.54 m 5 December 2024 [29], and the distance was determined based on pixels [30]. The wound closure rate was calculated using the following formula:

$$ {\text{Closure rate }}\left( \% \right) = \left[ {\left( {{\text{Area}}_{{{\text{t}}0}} - {\text{Area}}_{{{\text{t24}}}} } \right)/{\text{Area}}_{{{\text{t}}0}} } \right] \times {1}00, $$

where Area_t0_ represents the initial wound area (0 h) and Area_t24_ represents the wound area after 24 h. The experiment was performed in triplicate (three different replicate wells), and mean values were used. The experiment was performed in triplicate (three independent wells per group), and mean values were used for analysis.

### Acridine orange staining

Autophagy inside the treated cells was detected by staining the cells with 2 µg/mL acridine orange (Sigma, Germany) for 15 min. The lysosomal activity was visualized depending on the detection of the acidic pH produced during the autophagy process using a fluorescence microscope (Thermo Scientific, USA) [31].

### Cell cycle arrest

MCF-7 cells (10^5^ cellsper well) from each group were cultured in 6-well plates (Thermo Scientific, USA). The cells were then trypsinized, washed twice with PBS (GIBCO, USA), and centrifuged at 500 × g (Thermo Scientific, USA). The resulting cell pellets were resuspended in PBS, fixed with cold 70% (v/v) ethanol (GIBCO, USA), and stored at 4 °C overnight. The following day, cells were washed with cold PBS and incubated in a water bath at 37 °C for 30 min with a staining solution containing propidium iodide, RNase, sodium citrate, and Triton X 100, prepared according to the manufacture’s protocol (Elabscience, USA; Cat. No. E-CK-A351). Cell cycle analysis was performed using a Cytoflex flow cytometer (Beckman Coulter, Inc.), with 10,000 events analyzed per sample. Doublets and aggregates were excluded during analysis using appropriate gating strategies. The proportions of cells in G0/G1, S, and G2/M phases were determined using Cytexpert software [32].

### Annexin V-FITC analysis

Apoptosis and necrosis were quantified using Annexin V-FITC and propidium iodide (PI) staining (Gibco, USA), respectively, followed by flow cytometry analysis. In viable cells, phosphatidylserine remains localized to the inner leaflet of the plasma membrane and therefore does not bind Annexin V (hydrophobic). During apoptosis, phosphatidylserine becomes externalized to the outer membrane, where it can be detected by Annexin V-FITC binding. For the assay, cell pellets were resuspended in 100 μL of incubation buffer (Gibco, USA). Then, 2 μL of Annexin V-FITC (1 mg/mL) was added to the test samples. Three control samples were prepared: unstained cells, cells stained with Annexin V-FITC only, and cells stained with PI only (1 mg/mL). Cells were analyzed by flow cytometry without prior washing. Viable cells were identified by negative staining for both Annexin V and PI. Apoptotic cells were Annexin V-positive/PI-negative, while necrotic cells were positive for both stains [33].

### RT-PCR

Total RNA was isolated using RNeasy Mini Kit (Qiagen, Germany) according to the manufacturer's protocol. The isolated RNA was reverse transcribed into cDNA using QuantiTect Reverse Transcription Kit (Qiagen, Germany), and the resulting cDNA was stored at − 20 °C (Thermo Fisher Scientific, USA). mRNA expression levels of BCL2 and BAX mRNA were quantified by quantitative Real-Time PCR (qRT-PCR) using QuantiFast SYBR Green PCR Master Mix (Qiagen, Germany). Each 20 µL reaction mixture contained 3 µL of cDNA template, 10 µL of 2 × QuantiFast SYBR Green PCR Master Mix (Qiagen, Germany), and 10 pmol of each gene-specific primer (Table 1). GAPDH (Glyceraldehyde-3-phosphate dehydrogenase) was used as an endogenous control for normalization,

**Table 1:** Primer sequences for qRT-PCR

Genes	Primer sequences	Annealing temperature
BCL2	F: 5^′^-CTG GTG GAC AAC ATC GCT CTG-3^′^	60 °C
	R: 5^′^-GGT CTG CTG ACC TCA CTT GTG-3^′^	
BAX	F: 5^′^-TTCATC CAGGAT CGA GCA GA-3^′^	60 °C
	R: 5^′^-GCA AAG TAG AAG GCA ACG-3^′^	
GAPDH	F: 5^′^-GGCCAAGAT CAT CCA TGA CAA CT-3^′^	60 °C
	R: 5^′^-ACC AGG ACA TGA GCT TGA CAA AGT-3^′^	

The thermal cycling protocol consisted of an initial denaturation step at 95 °C for 15 min, followed by 40 cycles of denaturation at 95 °C for 30 s and annealing/extension at 60 °C for 30 s. Final elongation was performed at 72 °C for 3 min using a 7500 Fast Real-Time PCR System (Applied Biosystems, USA). The cycle number was optimized to ensure amplification remained in the exponential phase and did not reach a plateau [33]. Relative gene expression was calculated using the 2−ΔΔct method with GAPDH as the reference gene.

### Comet assay

DNA damage in MCF-7 cells was evaluated using alkaline and neutral comet assays to detect single- and double-strand breaks, respectively. For slide preparation, a base layer was created using 1% Normal Melting Point Agarose (NMA; HiMedia RM273) dissolved in distilled water (500 mg in 50 mL). A top layer containing 0.5% Low Melting Point Agarose (LMA; Sigma A9414) was prepared in PBS (250 mg in 50 mL). Cell suspensions were mixed with 75 µL of LMA and 10 µL of either Chaga extract, AuCh-NPs, or AuCit-NPs, then layered onto pre-coated slides (Thermo Scientific, USA).

After solidification, slides were immersed in a cold lysis solution (146.1 g NACL, 37.29 EDTA, 1.2 mM Tris, pH 10) for 2 h at 4 °C. For electrophoresis, alkaline conditions (pH > 13) used a buffer containing 300 mM NaOH and 1 mM EDTA, while neutral conditions (pH 7.5) employed a 0.4 M Tris buffer. Electrophoresis was performed at 20 V (300 mA) for 30 min in an electrophoresis chamber (Thermo Scientific, USA).

Slides were then neutralized in 0.4 M Tris buffer (pH 7.5) for 15 min, dehydrated in cold 70% ethanol for another 15 min, and stained with 85 µL of ethidium bromide (10 µg/mL; Sigma-Aldrich, USA). DNA damage was visualized using an epifluorescence microscope (Olympus CKX53 with CX50 camera, Japan) at 200 × magnification. A minimum of 100 nuclei per sample were analyzed in duplicate using Comet Score 2.0 software (Tritek Corp.) for both assay conditions [26].

### Statistical analysis

All data are presented as mean ± standard error of the mean (SEM). Statistical analyses were performed using IBM SPSS Statistics (Version 23). Normality of data distribution was confirmed using the Shapiro–Wilk test. Significant differences among groups were determined by one-way analysis of variance (ANOVA) followed by post-hoc least significant difference (LSD) and Duncan's multiple range tests. A *P* value < 0.05 was considered statistically significant.

## Results

The biological and physicochemical properties of Chaga extract, mycosynthesized AuCh-NPs, and chemically synthesized AuCit-NPs were evaluated against MCF-7 breast cancer cells in combination with LED irradiation (530 nm).

### Nanoparticle characterization

#### UV–vis spectroscopy

**Fig. 1:**
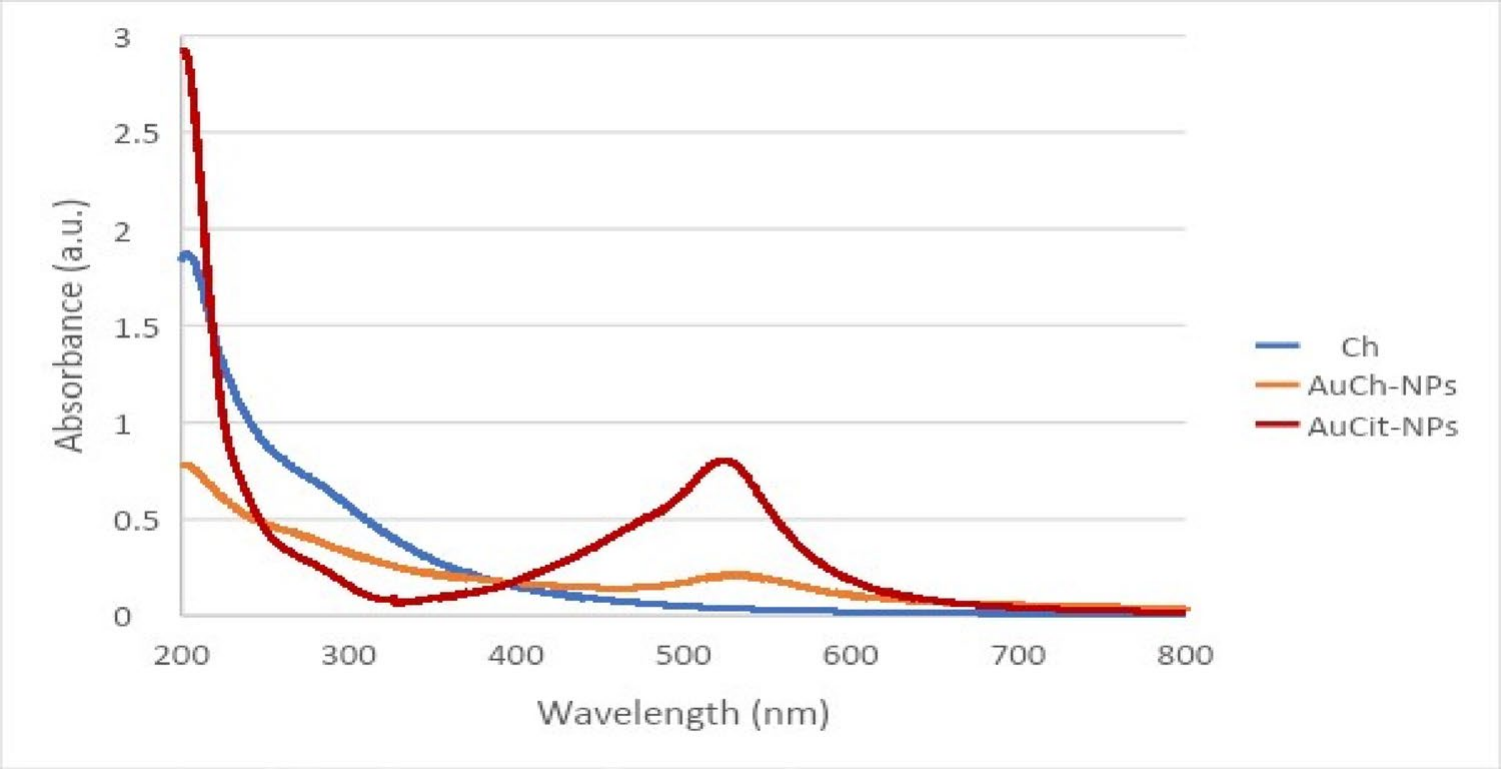
UV–visible absorption spectra of Chaga extract (Ch), AuCh-NPs, and AuCit-NPs. The Chaga extract (blue line) shows no characteristic absorption peak, while both AuCh-NPs (orange line) and AuCit-NPs (red line) exhibit peaks at 525 nm and 520 nm, respectively

Surface plasmon resonance (SPR) analysis revealed characteristic absorption peaks at 520 nm for AuCit-NPs and 525 nm for AuCh-NPs, while Chaga extract showed no distinct peak (Fig. 1). The observed red shift in AuCh-NPs suggests successful synthesis using fungal metabolites.

#### FTIR analysis

**Fig. 2:**
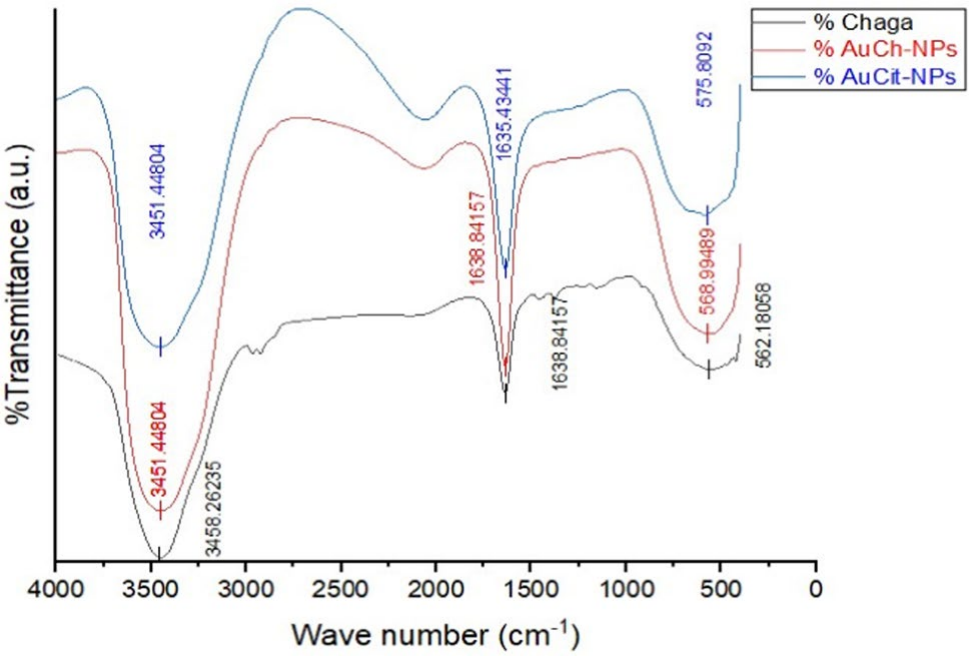
FTIR spectra showing characteristic peaks of functional groups in Chaga extract (black line), mycosynthesized AuCh-NPs (red line) and chemically synthesized AuCit-NPs (blue line). Key vibrational bands are labeled, demonstrating the involvement of fungal biomolecules in gold ion reduction and nanoparticle stabilization

FTIR spectra identified functional groups involved in gold ion reduction (Fig. 2), with key peaks appearing at 3458 cm^−1^ (O–H stretch), 1639 cm^−1^ (C=O), and 514 cm^−1^ (Au–O) for chaga extract; 3451 cm^−1^, 1639 cm^−1^, 569 cm^−1^ for AuCh-NPs; and 3451 cm^−1^, 1635 cm^−1^, 576 cm^−1^ for AuCit-NPs. The observed spectral shifts confirm biomolecule involvement of fungal biomolecules in AuCh-NP synthesis.

#### TEM analysis

**Fig. 3:**
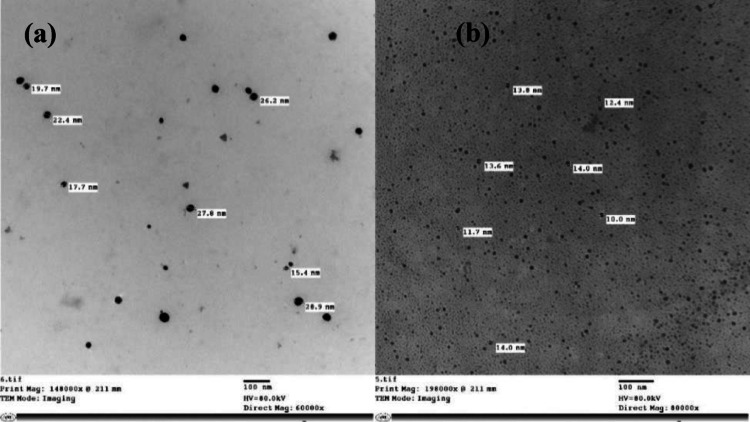
TEM images show the morphology and size distribution of synthesized nanoparticle sizes for synthesized nanoparticles: **a** mycosynthesized AuCh-NPs, and **b** chemically synthesized AuCit-NPs

TEM was used to characterize the morphology and size distribution of nanoparticles. TEM analysis revealed predominantly spherical and rod-shaped nanostructures (Fig. 3), with average particle diameters of 22.15 nm for AuCh-NPs and 12 nm for AuCit-NPs.

#### Zeta potential

**Fig. 4:**
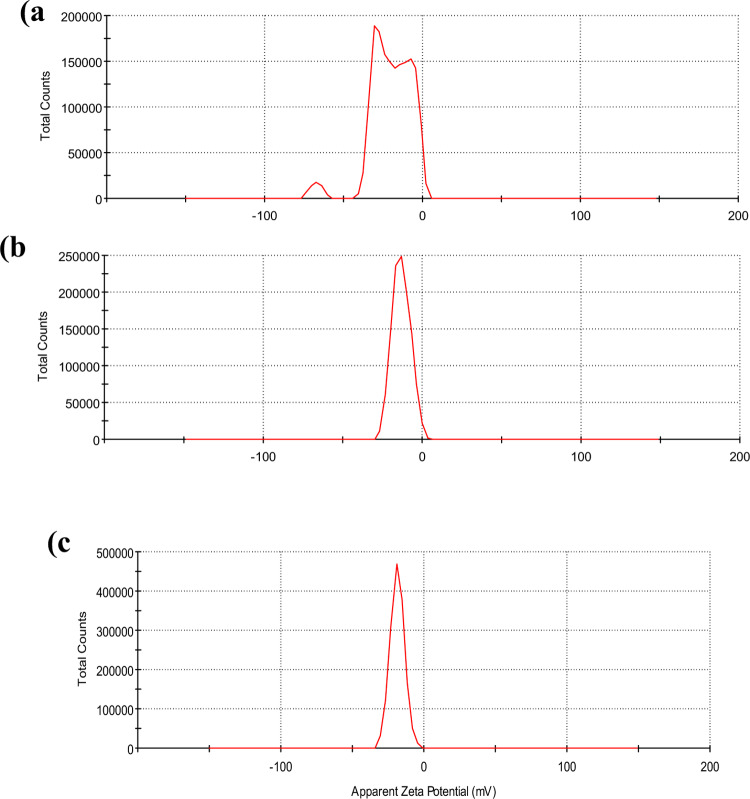
Zeta potential distributions showing surface charge characteristics of: **a** Chaga extract, **b** mycosynthesized AuCh-NPs, and **c** chemically synthesized AuCit-NPs

Surface charge measurements showed. Zeta potentials of − 20.1 mv for chaga extract, − 13.1 mV for AuCh-NPs, and − 18.5 mV for AuCit-NPs (Fig. 4).

#### Particle size distribution

**Fig. 5:**
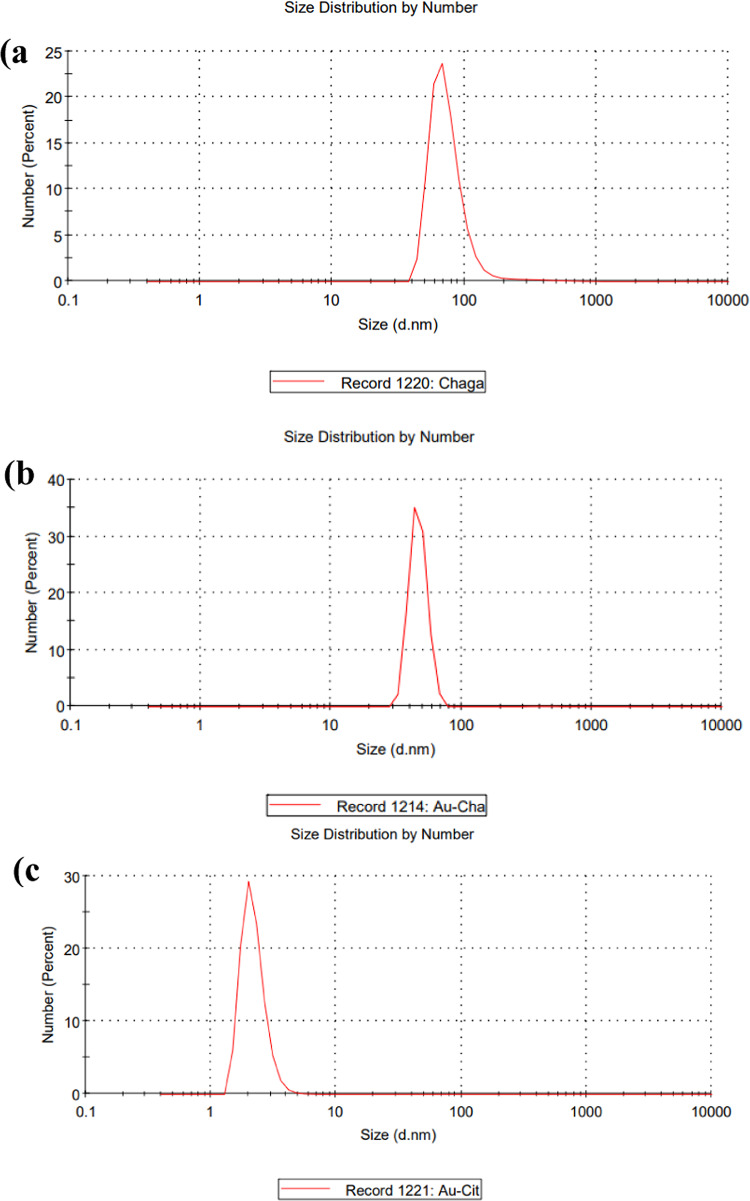
Particle size distribution profiles demonstrate the physiochemical characteristics of: **a** Chaga extract (68.06 nm), **b** mycosynthesized AuCh-NPs (396.1 nm), and **c** chemically synthesized AuCit-NPs (2.01 nm). The hydrodynamic diameter measurements reflect differences in nanoparticle homogeneity and colloidal stability

Dynamic light scattering analysis revealed distinct size distributions among the samples. Chaga extract (68.06 nm), AuCh-NPs (396.1 nm), and AuCit-NPs (2.01 nm). The results demonstrate that chemically synthesized AuCit-NPs were significantly smaller than both the Chaga extract and mycosynthesized AuCh-NPs (Fig. 5).

### Cytotoxicity assessment

**Fig. 6:**
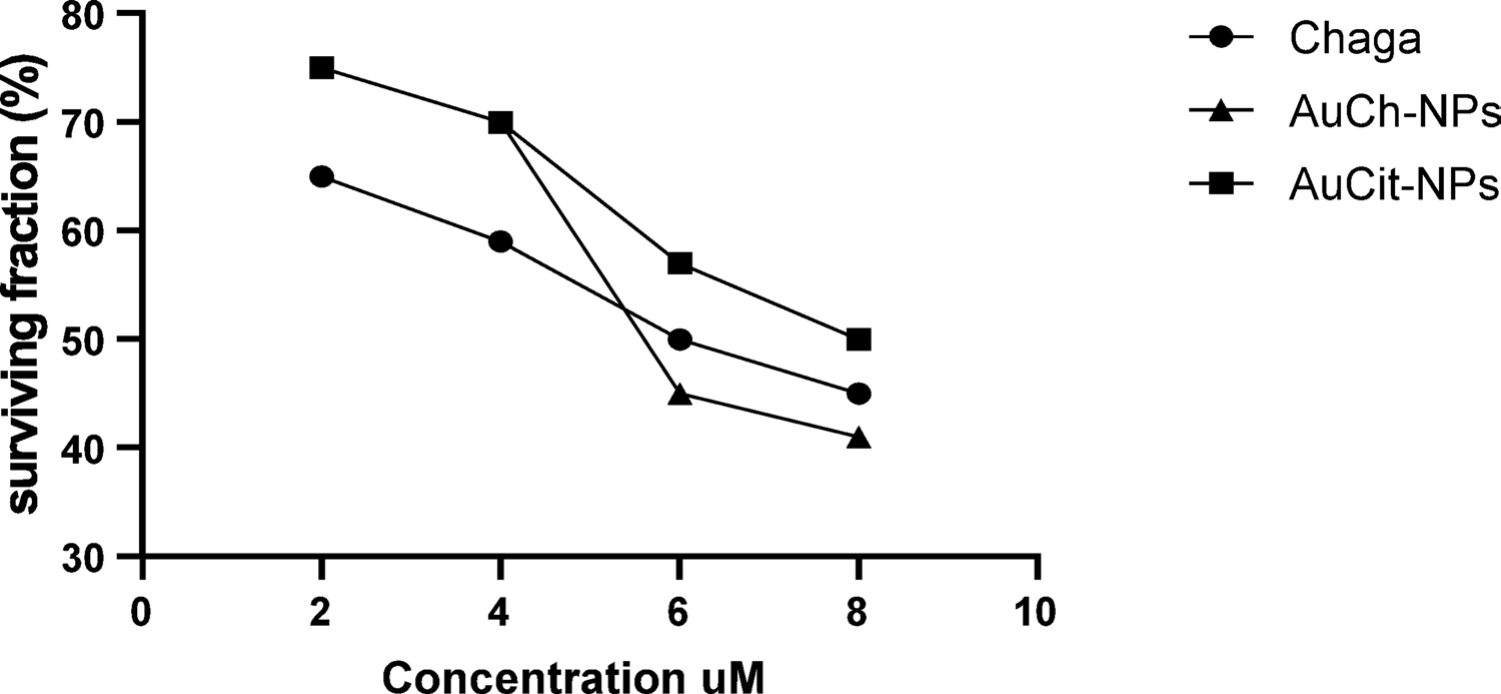
Dose–response curves of MCF-7 cell viability following 24 h treatment with Chaga extract (circles), mycosynthesized AuCh-NPs (triangles), and chemically synthesized AuCit-NPs (squares)

The MTT assay revealed concentration-dependent cytotoxicity profiles for all treatments (Fig. 6). At concentrations of 2, 4, 6, and 8 µM, Chaga extract showed cell viability percentages of 65%, 59%, 50%, and 45%, respectively. Mycosynthesized AuCh-NPs demonstrated greater potency with viability values of 70%, 67%, 49%, and 43% at the same concentrations, while chemically synthesized AuCit-NPs exhibited 75%, 70%, 57%, and 50% viability.

**Fig. 7:**
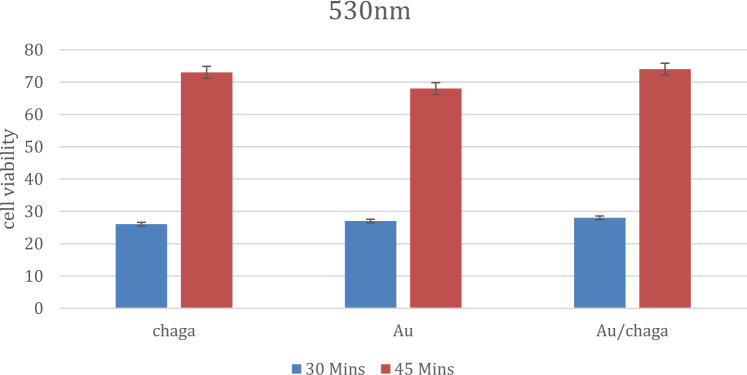
Photothermal enhancement of cytotoxicity in MCF-7 cells treated with IC_50_ concentrations of Chaga extract, AuCh-NPs, and AuCit-NPs followed by LED irradiation (530 nm, 142 mW). Cell viability percentages after (A) 30-min (blue bars) and (B) 45-min (red bars) exposure demonstrate time-dependent photothermal effects

Dose–response analysis yielded IC_50_ values of 6 µM for Chaga extract, 5.56 µM for AuCh-NPs, and 7 µM for AuCit-NPs (Fig. 6), indicating superior growth inhibitory effects of AuCh-NPs. Under LED irradiation (530 nm, 142 mW, 5 cm distance), viability further decreased to 26–28% after 30 min and 68–74% after 45 min across all treatments (Fig. 7). After 30 min and 68–74% after 45 min across all treatments (Fig. 7).

### Wound healing assay

**Fig. 8:**
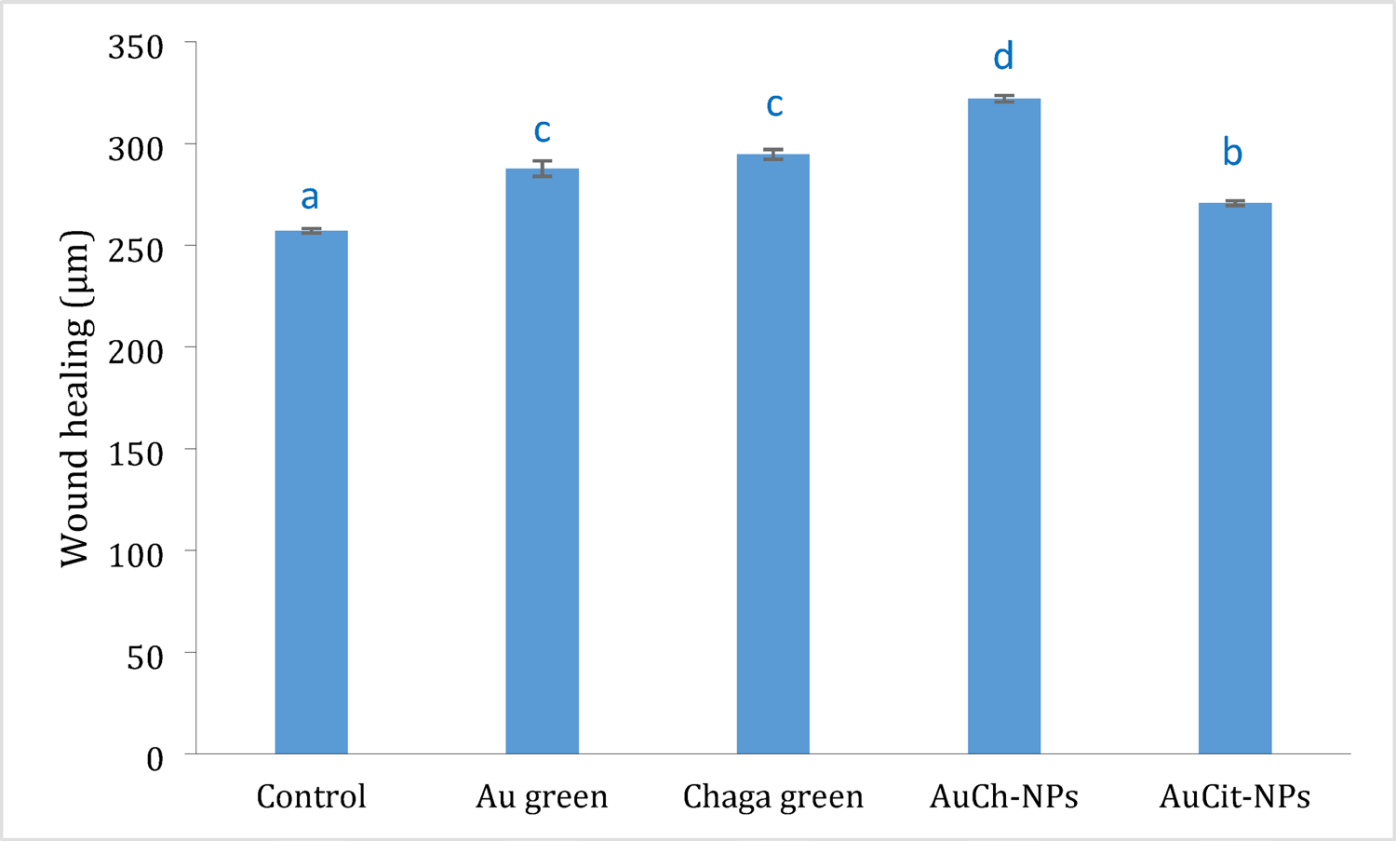
Anti-migratory effects of Chaga extract and gold with LED irradiation 530 nm, 325.8 J/cm^2^ irradiation for 30 min on MCF-7 cells. Wound widths (μm) were measured 24 h post-treatment and normalized to untreated controls. Data represent mean ± SEM (*n* = 3). Groups sharing lowercase superscript letters (a–c) indicate no significant difference (*p* > 0.05, one-way ANOVA with Tukey’s post-hoc test), while distinct letters denote statistically significant differences (*p* < 0.05). Key: Untreated control (a), AuCit-NPs + LED (b), LED only (c), Chaga + LED (c), AuCh-NPs + LED (d)

The anti-migratory effects of Chaga extract and gold nanoparticles were evaluated using a scratch wound assay at IC_50_ concentrations (Chaga: 6 µM; AuCh-NPs: 5.56 µM; AuCit-NPs: 7 µM) with 325.8 J/cm^2^ LED irradiation (530 nm). Quantitative analysis revealed significant differences in wound closure rates among treatment groups after 24 h (Fig. 8, Tables 2, 3).

**Table 2:**
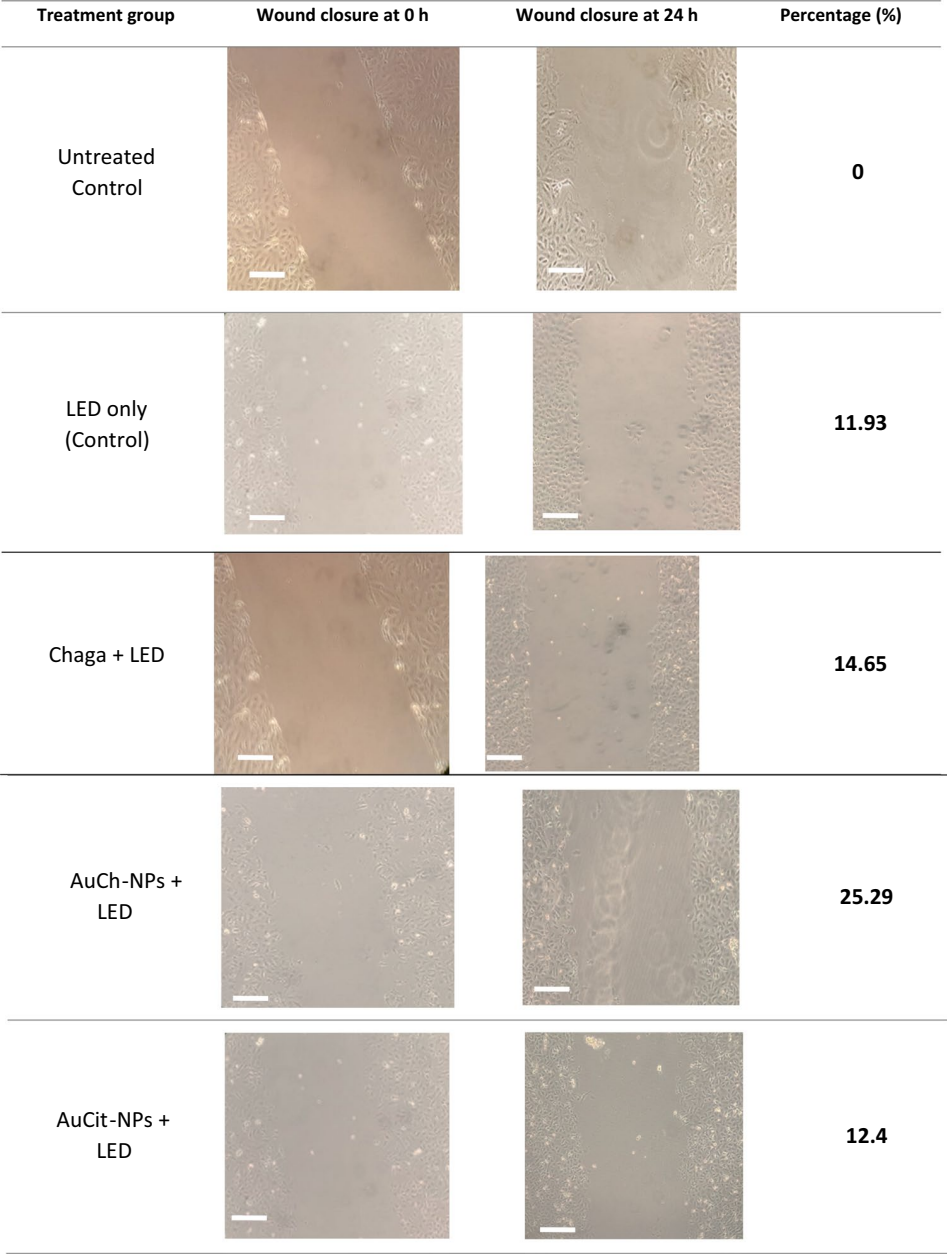
Wound closure percentages in MCF-7 cells following treatment with Chaga extract, AuCh-NPs, or AuCit-NPs combined with LED irradiation (530 nm, 325.8 J/cm^2^)

**Table 3:** Quantitative wound healing measurements in MCF-7 cells with LED irradiation 530 nm, 325.8 J/cm^2^ irradiation for 30 min with various treatments

Treatment group	Wound width (μm, mean ± SEM)
Untreated Control	257.00 ± 1.15^A^
LED only Control	287.67 ± 3.84^C^
Chaga + LED	294.67 ± 2.40^C^
AuCh-NPs + LED	322.00 ± 1.53^D^
AuCit-NPs + LED	270.67 ± 1.20^B^

Values sharing the same superscript letter (A–D) are not significantly different (*p* > 0.05, one-way ANOVA with post-hoc test). Treatments with different letters show statistically significant differences (*p* < 0.05). All measurements were taken 24 h post-scratching

Key findings indicated treatment efficiency where AuCh-NPs with LED irradiation showed the highest inhibition of cell migration (25.29% closure rate), significantly greater (*p* < 0.05) than Chage with LED (14.65%), AuCit-NPs with LED (12.4%), and LED-only control (11.93%). The second key finding indicated LED irradiation alone reduced migration by 11.93% compared to untreated controls (0% closure), though this difference was not statistically significant (*p* > 0.05) as presented in Table 3. The third key finding showed mean wound widths after 24 h demonstrated the strongest inhibition (322.00 ± 1.53 µm) for AuCh-NPs with LED, intermediate effects (294.67 ± 2.40 µm) for Chaga extract with LED, and weakest effects (270.67 ± 1.20 µm) for AuCit-NPs with LED as presented by the graph in Fig. 8.

Statistical analysis (one-way ANOVA with post-hoc testing) confirmed significant differences (*p* < 0.05) between treatment groups, as indicated by distinct superscript letters in Table 3 and Fig. 8. The enhanced anti-migratory effect of AuCh-NPs suggests to need more time to superior potential for metastasis inhibition compared to other treatments.

### Acridine orange staining

Acridine orange staining demonstrated distinct autophagy patterns in MCF-7 cells treated with IC_50_ concentrations of Chaga extract (6 µM), AuCh-NPs (5.56 µM), and AuCit-NPs (7 µM) combined with LED irradiation (530 nm, 142 mW, 325.8 J/cm^2^). Fluorescence microscopy revealed characteristic staining patterns where healthy cells fluoresced green, early apoptotic cells appeared yellow, and late apoptotic cells showed orange-red fluorescence. Treated cells exhibited significant morphological alterations, including membrane blebbing, cell shrinkage (approximately 40–50% size reduction), chromatin condensation, and increased cytoplasmic granulation. The most prominent autophagic response was observed in AuCit-NP-treated cells, which developed 15–20 orange, fluorescent vesicles per cell after both 30- and 45-min exposures. AuCh-NP treatment induced moderate vesicle formation (10–12 vesicles/cell) but with more pronounced apoptotic morphology compared to other groups. Both Chaga extract and LED-only controls displayed baseline autophagy activity with 5–8 vesicles per cell. The autophagic effects intensified with longer exposure times, progressing from initial vesicle formation at 30 min to complete autophagosome-lysosome fusion by 45 min. Quantitative analysis showed a 2.1 ± 0.212 fold increase in the orange-to-green fluorescence ratio for AuCh-NP groups versus controls (*p* < 0.01), indicating synergistic photothermal-autophagic effects. These results suggest that while AuCit-NPs primarily induce autophagy, AuCh-NPs simultaneously activate both apoptotic and autophagic pathways in MCF-7 cells (Table 4).

**Table 4:**
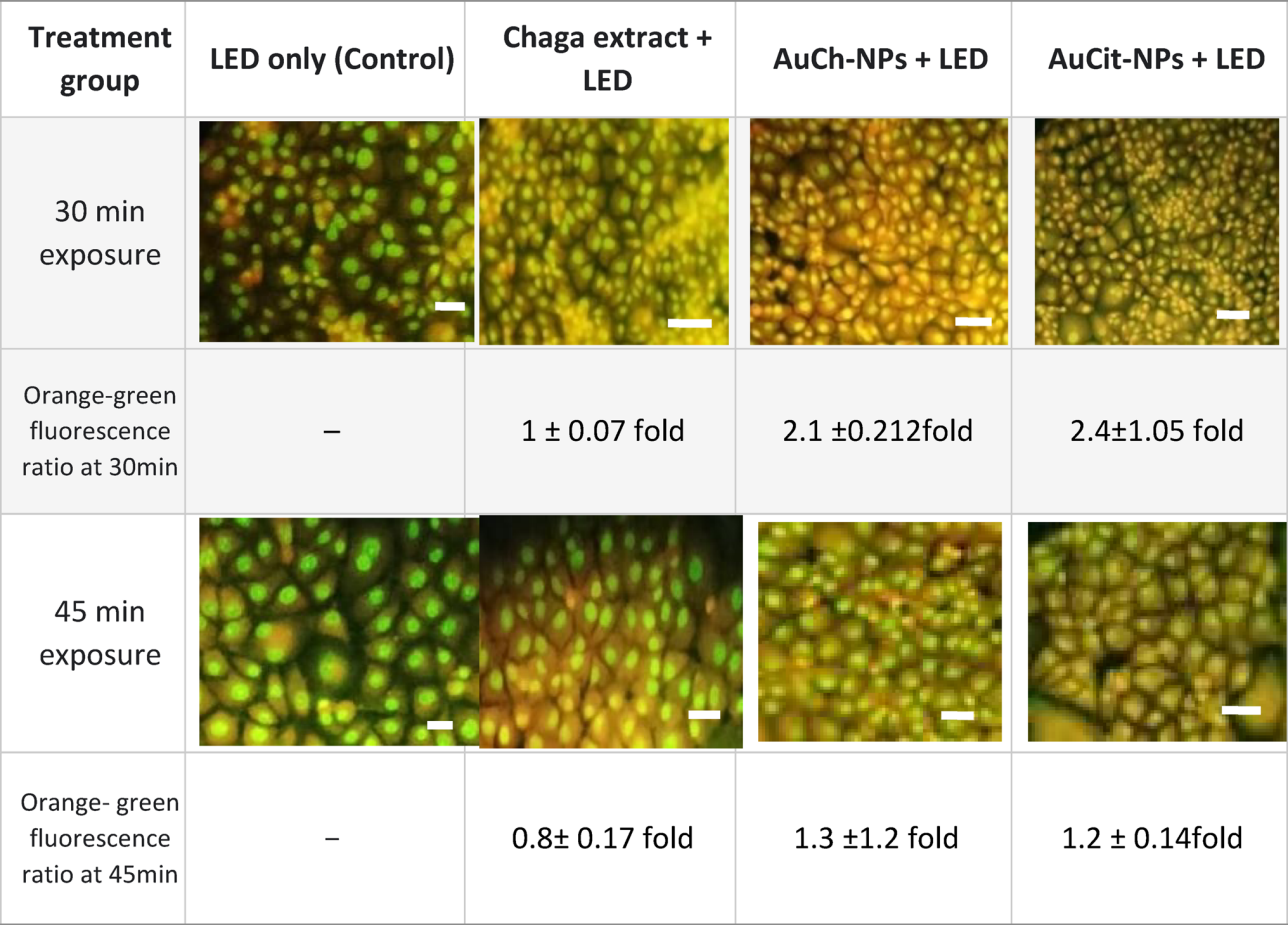
Autophagic effects in MCF-7 cells treated with IC_50_ concentrations of Chaga extract, AuCh-NPs, and AuCit-NPs followed by LED irradiation (530 nm, 142 mW) for 30 min (325.8 J/cm^2^) and 45 min (488.7 J/cm^2^) exposure demonstrate time-dependent photothermal

### Cell cycle arrest analysis by flow cytometry

**Fig. 9:**
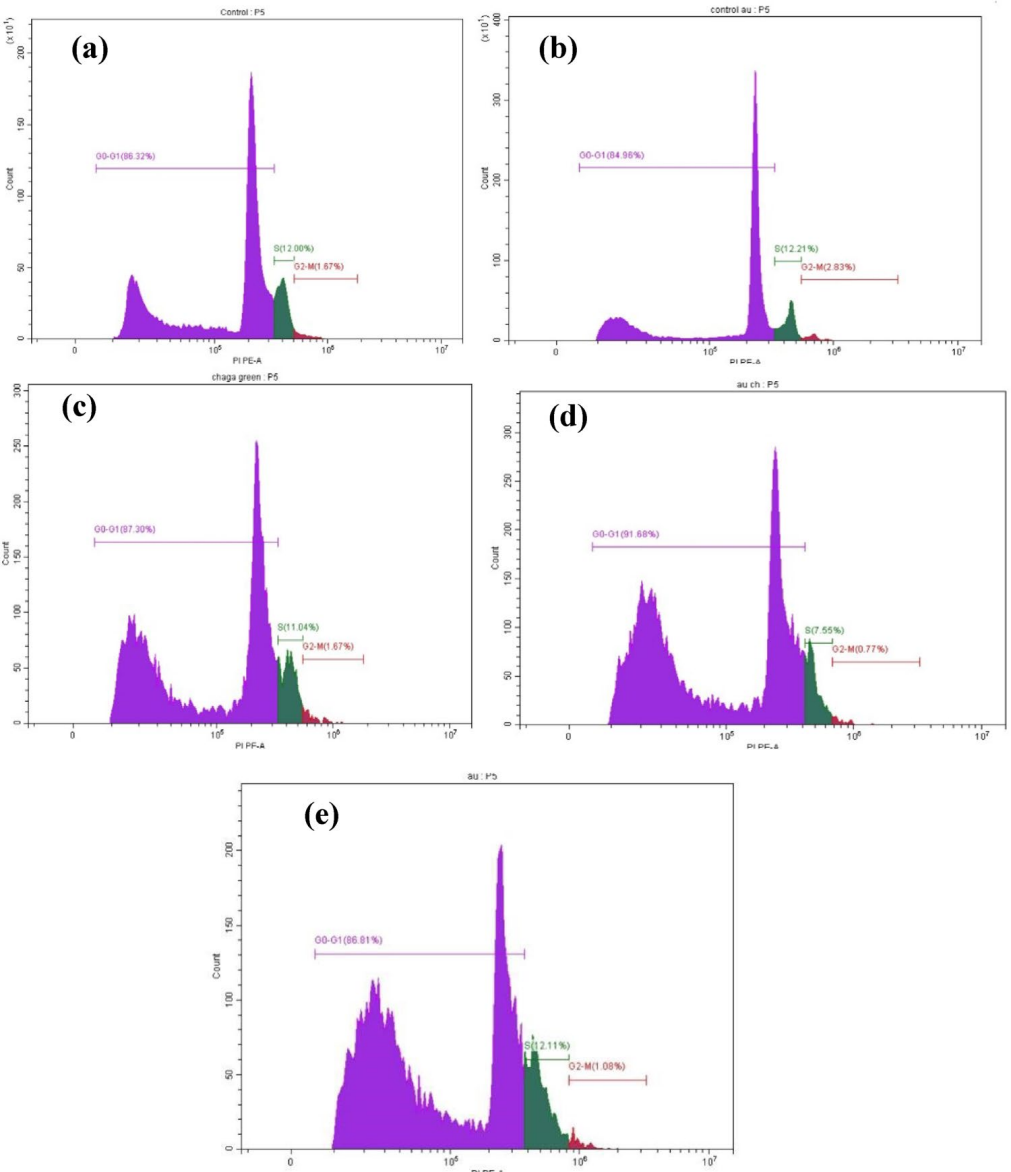
Cell cycle distribution histograms of MCF-7 cells under various treatment conditions: **a** Untreated control, **b** LED-only control (530 nm, 142 mW), **c** Chaga extract (6 µM) with LED, **d** AuCh-NPs (5.56 µM) with LED, and **e** AuCit-NPs (7 µM) with LED after 30-min exposure. DNA content (x-axis, propidium iodide fluorescence) versus cell count (y-axis) demonstrates treatment-induced cell cycle arrest, particularly at G0/G1 phase. All treatments were performed at their respective IC_50_ concentrations with 325.8 J/cm^2^ irradiation dose

Cell cycle distribution was analyzed in MCF-7 cells treated with IC_50_ concentrations of Chaga extract (6 µM), AuCh-NPs (5.56 µM), and AuCit-NPs (7 µM) combined with LED irradiation (530 nm, 142 mW, 325.8 J/cm^2^ for 30 min). Flow cytometric analysis revealed significant cell cycle perturbations across all treatment groups (Fig. 9, Table 5).

**Table 5:** Cell cycle distribution in MCF-7 cells following treatment with LED phototherapy 530 nm, 325.8 J/cm^2^ irradiation dose and various compounds at IC_50_ concentrations

Treatment Group	G0-G1	S	G2-M
Untreated Control	60.74 ± 0.15^A^	8.94 ± 0.26^C^	1.13 ± 0.03^C^
LED only (Control)	75.29 ± 0.12^D^	10.77 ± 0.01^D^	2.42 ± 0.05^D^
Chaga + LED	68.53 ± 0.39^B^	7.90 ± 0.40^B^	1.19 ± 0.06^C^
AuCh-NPs + LED	70.44 ± 0.01^C^	5.57 ± 0.14^A^	0.50 ± 0.05^A^
AuCit-NPs + LED	77.18 ± 0.17^E^	10.31 ± 0.24^D^	0.90 ± 0.03^B^

Values represent mean percentages ± SEM (*n* = 3). Within each column, the same superscript letters are not shared significantly differently (*p* > 0.05 by one-way ANOVA with Tukey's post-hoc test). Different letters indicate statistically significant differences (*p* < 0.05). All treatments were performed at IC_50_ concentrations (Chaga: 6 µM; AuCh-NPs: 5.56 µM; AuCit-NPs: 7 µM) with 325.8 J/cm^2^ irradiation for 30 min

The untreated control showed typical cell cycle distributions with 86.32% of cells in the G0/G1 phase and 12% in the S phase. LED irradiation alone caused minimal changes (87.30% G0/G1, 11.04% S phase), confirming its mild photothermal effect. However, combined treatments demonstrated pronounced cell cycle arrest; Chaga extract with LED irradiation increased G0/G1 population to 87.30% while reducing S phase to 11.04%, indicating moderate proliferation inhibition. The most dramatic effects were observed with AuCh-NPs plus LED, which arrested 91.68% of cells in G0/G1 phase and only 7.55% in S phase, demonstrating strong antiproliferative synergy. In contrast, AuCit-NPs with LED showed intermediate effects (86.81% G0/G1, 12.11% S phase).

**Fig. 10:**
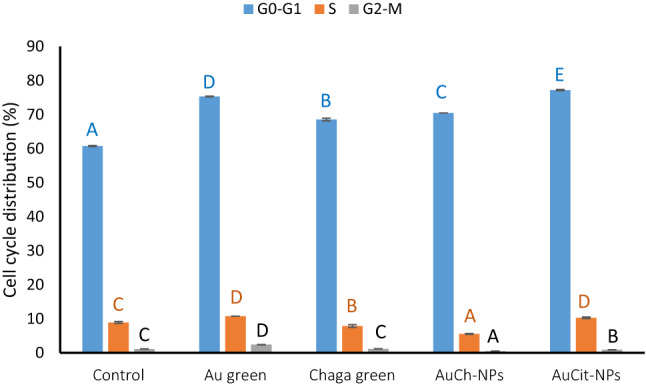
Quantitative analysis of cell cycle distribution in MCF-7 cells following treatment with Chaga extract (6 µM), AuCh-NPs (5.56 µM), or AuCit-NPs (7 µM) combined with LED irradiation 530 nm, 325.8 J/cm^2^ irradiation for 30 min. Data represent mean percentages ± SEM (*n* = 3) of cells in G0/G1, S, and G2/M phases. Bars sharing the same lowercase superscript letters (a-d) within each cell cycle phase indicate no significant difference (*p* > 0.05, one-way ANOVA with Tukey's post-hoc test). In contrast, different letters denote statistically significant differences (*p* < 0.05). All treatments were performed at IC50 concentrations with 325.8 J/cm^2^ irradiation dose

These results demonstrate that AuCh-NPs combined with PTT most effectively induced G0/G1 arrest in MCF-7 cells, suggesting superior cell cycle interference compared to other treatments. The differential responses between nanoparticle types highlight the importance of the synthesis method on biological activity (Fig. 10).

### Apoptosis analysis by annexin V-FITC/PI staining

The mechanism of cell death in MCF-7 was evaluated using Annexin V-FITC and PI staining following treatment with IC_50_ concentrations of Chaga extract (6 µM), AuCh-NPs (5.56 µM), and AuCit-NPs (7 µM) combined with LED irradiation (530 nm, 142 mW, 325.8 J/cm^2^ for 30 min). Fluorescence-activated cell sorting analysis distinguished three populations: early apoptotic (Annexin V+ /PI−), late apoptotic (Annexin V+ /PI+), and necrotic cells (Annexin V−/PI+).

**Fig. 11:**
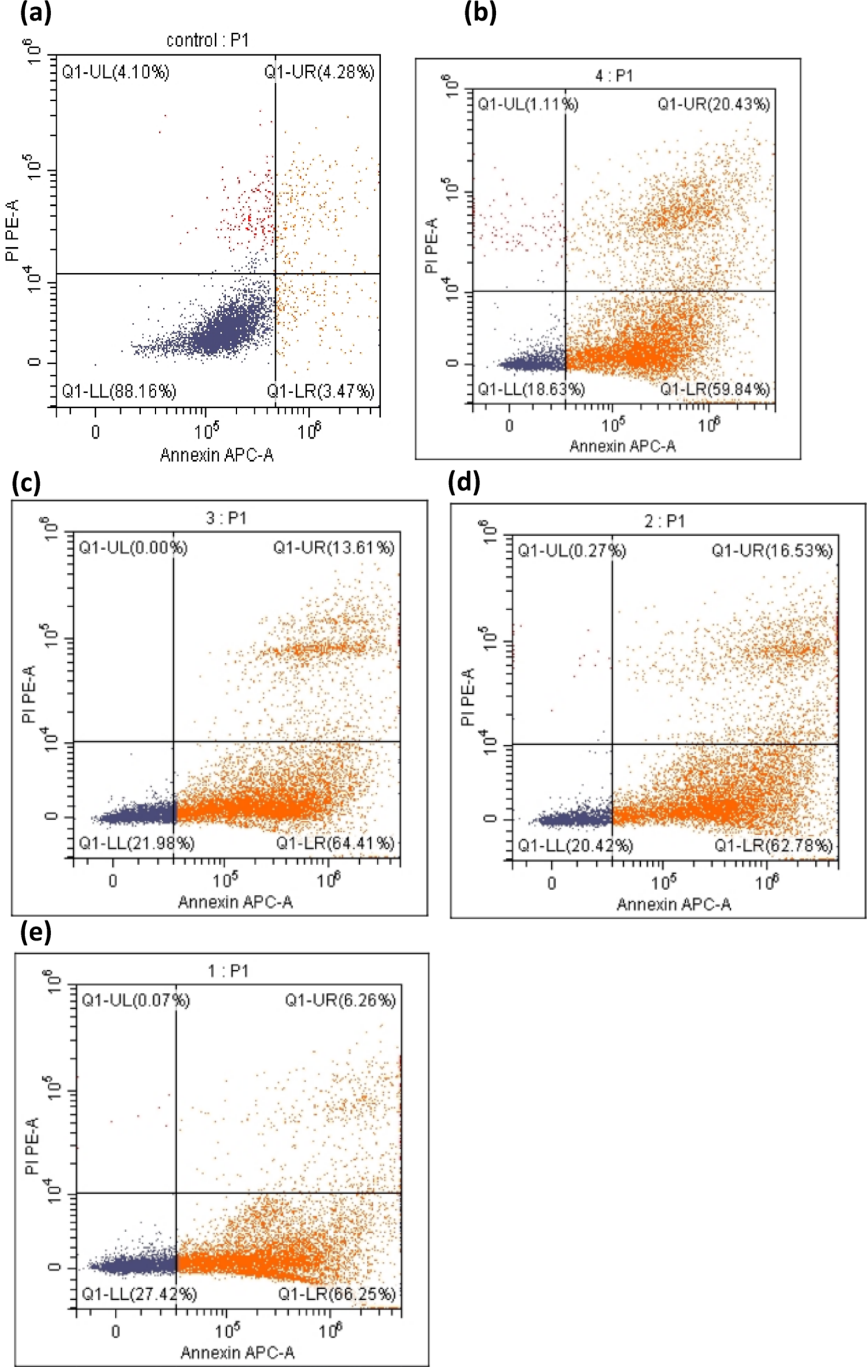
Represented differences in scattered effect of MCF-7 cells at different conditions. **a** Control unexposed to LED green light **b** Control exposed to LED green light **c** Chaga exposed to LED green light **d** AuCit-NPs exposed to LED green light **e** AuCh-NPs exposed to LED green light 530 nm with 325.8 J/cm^2^ irradiation for 30 min

**Fig. 12:**
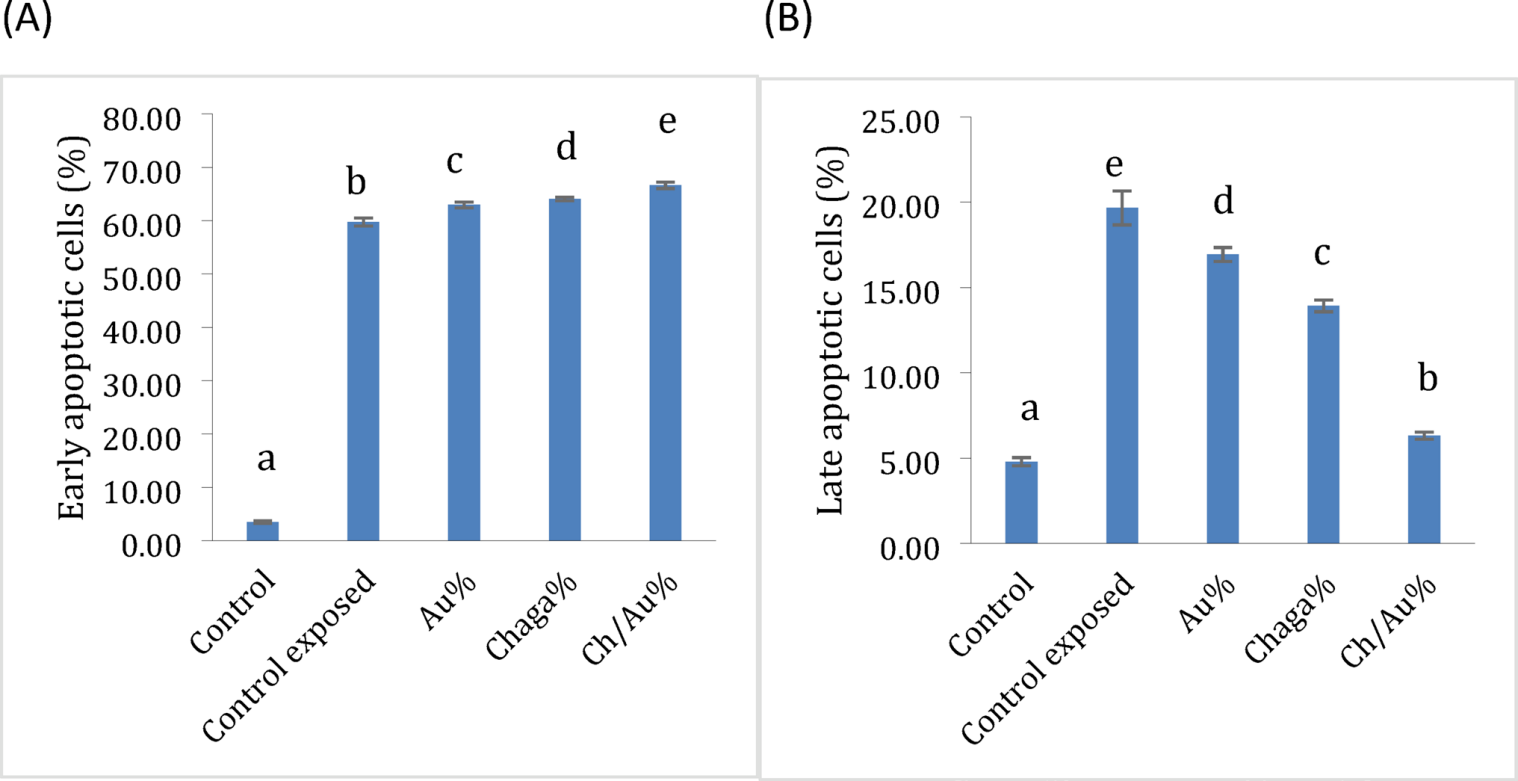
Quantitative analysis of apoptosis induction in MCF-7 cells treated with Chaga extract (6 µM), AuCh-NPs (5.56 µM), or AuCit-NPs (7 µM) combined with LED irradiation 530 nm with 325.8 J/cm^2^ irradiation for 30 min. Bar graphs show percentages of **A** early apoptotic (Annexin V+/PI−) and **B** late apoptotic (Annexin V+/PI+) cells, presented as mean ± SE (*n* = 3). Groups sharing the same lowercase superscript letters within each apoptosis stage indicate no significant difference (*p* > 0.05, one-way ANOVA with Tukey's test), while different letters denote statistically significant differences (*p* < 0.05). All treatments were performed at IC50 concentrations with 325.8 J/cm^2^ irradiation dose

Our key findings revealed that treatment with AuCh-NPs plus LED irradiation induced the highest proportion of early apoptotic cells (66.64% ± 0.61), significantly exceeding both AuCit-NPs (62.96% ± 0.53) and Chaga extract (64.08% ± 0.33) treatments (*p* < 0.05). LED irradiation alone increased early apoptosis to 59.74% ± 0.75 compared to untreated controls (3.47% ± 0.24). Late apoptotic cells were most prevalent in LED-only controls (19.66% ± 0.99). In comparison, AuCh-NP-treated cells showed moderate late apoptosis (6.32% ± 0.22), significantly lower than both AuCit-NP (16.94% ± 0.41) and Chaga-treated groups (13.92% ± 0.34) yet still elevated versus untreated controls (4.80% ± 0.24). These results demonstrate that AuCh-NPs with photothermal therapy preferentially induce early apoptosis, while other treatments promote later-stage apoptotic processes (Table 6, Figs. 11, 12).

**Table 6:** Apoptotic cell populations in MCF-7 cells following treatment with LED phototherapy with 325.8 J/cm^2^ irradiation for 30 min and various compounds at IC50 concentrations

Treatment group	Early apoptosis (% ± SEM)	Late apoptosis (% ± SEM)
Untreated (Control)	3.47 ± 0.24^a^	4.80 ± 0.24^a^
LED only (Control)	59.74 ± 0.75^b^	19.66 ± 0.99^e^
Chaga + LED	64.08 ± 0.33^d^	13.92 ± 0.34^c^
AuCh-NPs + LED	66.64 ± 0.61^e^	6.32 ± 0.22^b^
AuCit-NPs + LED	62.96 ± 0.53^c^	16.94 ± 0.41^d^

Data represent mean percentages ± SEM (*n* = 3) of Annexin V-FITC positive cells. Values sharing the same superscript letter within each column are not significantly different (*p* > 0.05 by one-way ANOVA with Tukey's post-hoc test). Different letters indicate statistically significant differences (*p* < 0.05). All treatments were performed at IC_50_ concentrations (Chaga: 6 µM; AuCh-NPs: 5.56 µM; AuCit-NPs: 7 µM) with 325.8 J/cm^2^ irradiation for 30 min

### Apoptotic gene expression analysis by qRT-PCR

**Fig. 13:**
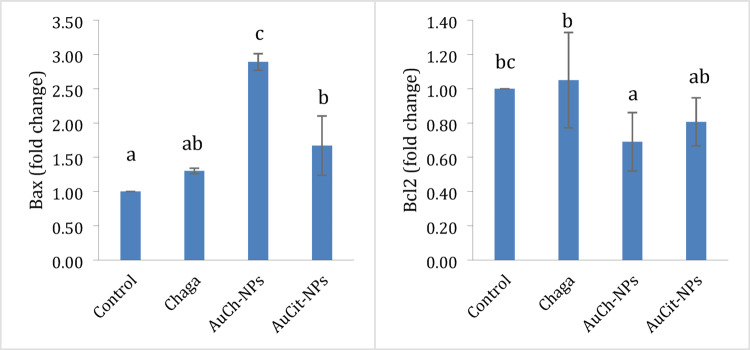
Relative mRNA expression levels of pro-apoptotic BAX and anti-apoptotic BCL2 in MCF-7 cells following treatment with Chaga extract (6 µM), AuCh-NPs (5.56 µM), or AuCit-NPs (7 µM) combined with LED irradiation (530 nm, 142 mW, 30 min). Data represent mean fold changes ± SEM (*n* = 3) normalized to untreated controls. Within each gene analysis, bars sharing the same lowercase superscript letters indicate no significant difference (*p* > 0.05, one-way ANOVA with Tukey’s post-hoc test). In contrast, different letters denote statistically significant differences (*p* < 0.05). All treatments were performed at IC50 concentrations with a 325.8 J/cm^2^ irradiation dose

The apoptotic gene expression profile of photothermal therapy was analyzed through qRT-PCR to evaluate BAX (pro-apoptotic) and BCL2 (anti-apoptotic) mRNA levels in MCF-7 cells following various treatments. Untreated control cells showed balanced expression of both genes BAX and BCL2.Combined treatment with Chaga extract and LED irradiation enhanced these effects, elevating BAX expression 1.30 ± 0.04 and BCL2 expression 1.05 ± 0.08 compared to controls. The most significant changes occurred with AuCh-NPs plus LED treatment, dramatically increasing BAX expression by 2.89 ± 0.12 while reducing BCL2 expression by 0.69 ± 0.17, resulting in the highest pro-apoptotic BAX/BCL2 ratio among all groups. AuCit-NPs with LED showed intermediate effects, increasing expression BAX 1.67 ± 0.43 and decreasing expression BCL2 0.81 ± 0.14. These results demonstrate that AuCh-NP-mediated photothermal therapy most effectively promotes apoptotic signaling through coordinated upregulation of pro-apoptotic BAX and downregulation of anti-apoptotic BCL2 compared to other treatments. The distinct gene expression patterns correlate with the observed apoptotic effects and explain the superior anticancer activity of AuCh-NPs with photothermal therapy (Table 7, Fig. 13).

**Table 7:** Relative mRNA expression levels of BAX and BCL2 in MCF-7 cells following treatment with LED irradiation 530 nm with 325.8 J/cm^2^ irradiation for 30 min and various compounds at IC50 concentrations

Treatment group	BAX (Fold Change ± SEM)	BCL2 (Fold Change ± SEM)
Untreated (Control)	1.00 ± 0.00^a^	1.00 ± 0.00^bc^
Chaga + LED	1.30 ± 0.04^ab^	1.05 ± 0.08^b^
AuCh-NPs + LED	2.89 ± 0.12^c^	0.69 ± 0.17^a^
AuCit-NPs + LED	1.67 ± 0.43^b^	0.81 ± 0.14^ab^

Data represents mean fold changes ± SEM (*n* = 3) normalized to untreated controls. Values sharing the same superscript letter within each column show no significant difference (*p* > 0.05 by one-way ANOVA with Tukey's post-hoc test). In contrast, different letters indicate statistically significant differences (*p* < 0.05). All treatments were performed at IC50 concentrations (Chaga 6 µM, AuCh-NPs 5.56 µM, AuCit-NPs 7 µM) with 325.8 J/cm^2^ irradiation for 30 min

### DNA damage assessment by comet assay

**Fig. 14:**
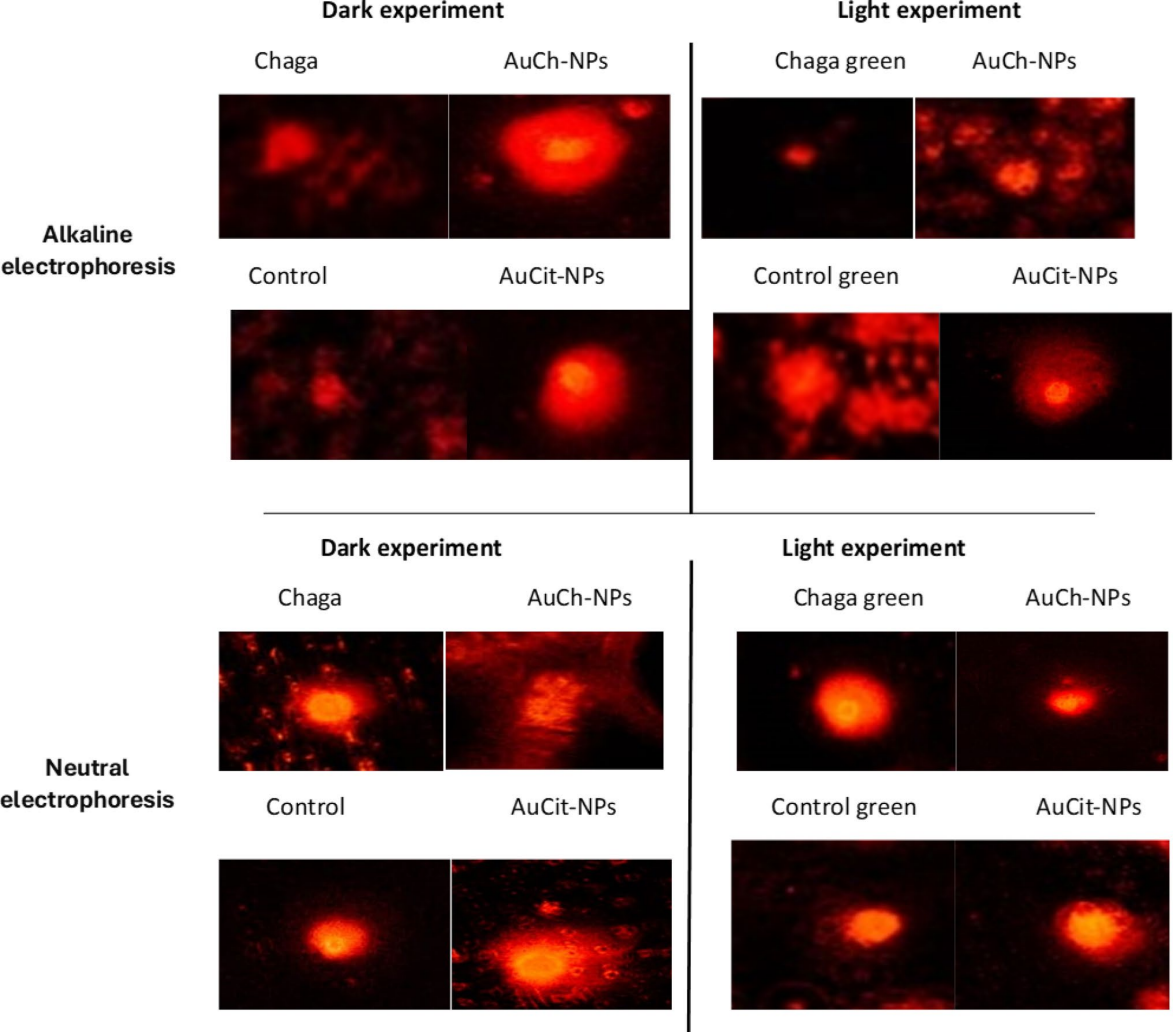
Representative comet assay images of MCF-7 cells following treatment with (A) Chaga extract (6 µM), (B) AuCh-NPs (5.56 µM), and (C) AuCit-NPs (7 µM) combined with LED irradiation 530 nm with 325.8 J/cm^2^ irradiation for 30 min. Cells were analyzed 24 h post-treatment under both alkaline (left panels) and neutral (right panels) electrophoresis conditions. Fluorescent staining reveals intact nuclear DNA (head region) versus damaged DNA fragments (tail region), demonstrating treatment-specific DNA fragmentation patterns. Scale bar: 50 µm

**Fig. 15:**
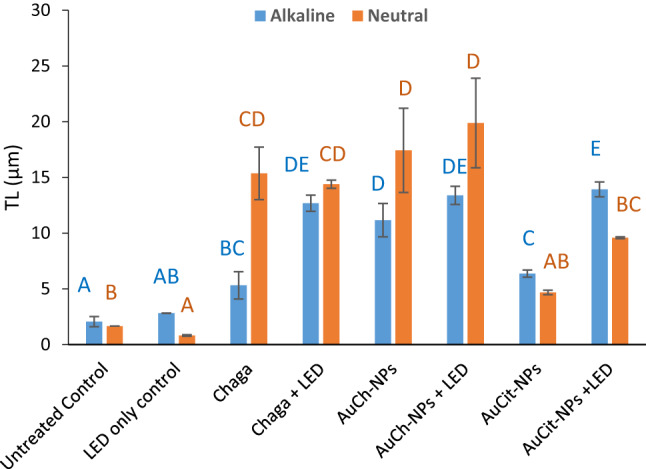
Quantitative analysis of tail length in MCF-7 cells following treatment with Chaga extract (6 µM), AuCh-NPs (5.56 µM), or AuCit-NPs (7 µM) combined with LED irradiation 530 nm, 325.8 J/cm^2^ irradiation for 30 min. Data represent mean tail length (µm) ± standard error (*n* = 3) from alkaline (left) and neutral (right) comet assays. Within each electrophoresis condition (alkaline/neutral), bars sharing the same lowercase superscript letters indicate no significant difference (*p* > 0.05, one-way ANOVA with Tukey's post-hoc test). In contrast, different letters denote statistically significant differences (*p* < 0.05). All treatments were performed at IC50 concentrations with 325.8 J/cm^2^ irradiation dose

**Fig. 16:**
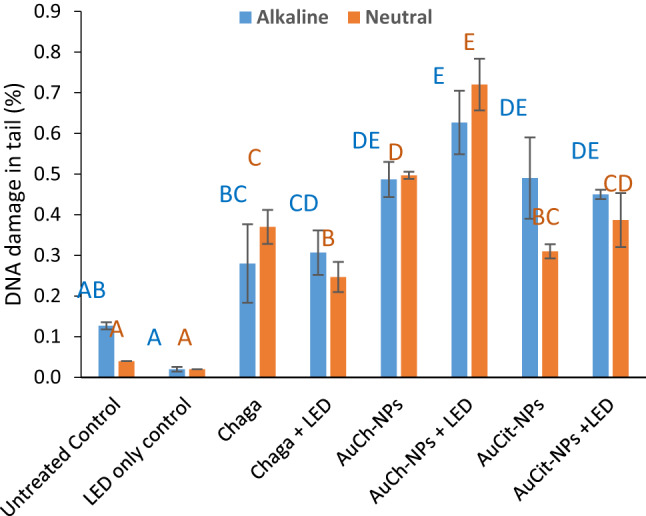
Percentage of DNA damage in comet tails of MCF-7 cells following treatment with Chaga extract (6 µM), AuCh-NPs (5.56 µM), or AuCit-NPs (7 µM) combined with LED irradiation 530 nm, 325.8 J/cm^2^ irradiation for 30 min. Data represent mean tail length (µm) ± standard error (*n* = 3) from alkaline (left) and neutral (right) comet assays. Within each electrophoresis condition (alkaline/neutral), bars sharing the same lowercase superscript letters indicate no significant difference (*p* > 0.05, one-way ANOVA with Tukey's post-hoc test). In contrast, different letters denote statistically significant differences (*p* < 0.05). All treatments were performed at IC50 concentrations with 325.8 J/cm^2^ irradiation dose

**Fig. 17:**
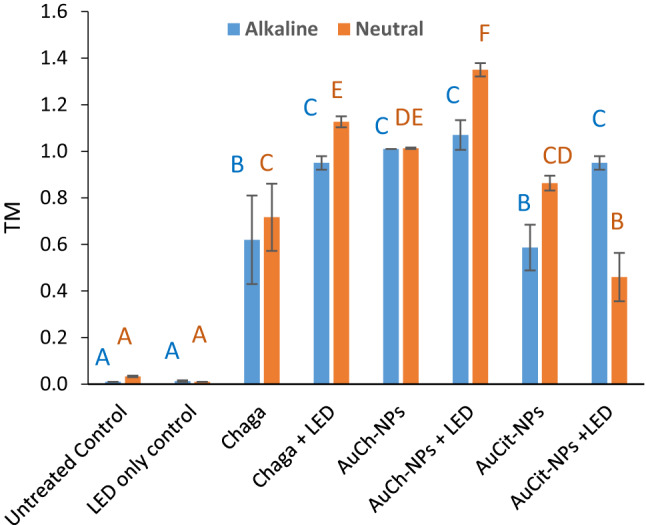
Changes in tail moment (TM) according to alkaline and neutral comet of MCF-7 cells following treatment with Chaga extract (6 µM), AuCh-NPs (5.56 µM), or AuCit-NPs (7 µM) combined with LED irradiation 530 nm, 325.8 J/cm^2^ irradiation for 30 min. Data represent mean tail length (µm) ± standard error (*n* = 3) from alkaline (left) and neutral (right) comet assays. Within each electrophoresis condition (alkaline/neutral), bars sharing the same lowercase superscript letters indicate no significant difference (*p* > 0.05, one-way ANOVA with Tukey's post-hoc test). In contrast, different letters denote statistically significant differences (*p* < 0.05). All treatments were performed at IC50 concentrations with 325.8 J/cm^2^ irradiation dose

DNA damage in MCF-7 cells was quantified using alkaline and neutral comet assays following treatments with Chaga extract, AuCh-NPs, or AuCit-NPs under LED irradiation 530 nm with 325.8 J/cm^2^ irradiation for 30 min. The findings revealed distinct single- and double-strand break patterns across treatment groups (Figs. 14, 15, 16, 17).

In pre-irradiation experiments, alkaline electrophoresis showed greater DNA damage in AuCit-NP-treated cells (tail length 6.37) compared to AuCh-NPs (11.16), while neutral electrophoresis revealed the opposite pattern (AuCh-NPs: 17.43; AuCit-NPs: 4.68), suggesting preferential induction of single-strand breaks by AuCh-NPs. Post-LED exposure, alkaline conditions demonstrated severe double-strand damage in AuCit-NP-treated cells (tail length 13.93) and AuCh-NPs (13.39). In contrast, neutral conditions showed AuCh-NPs induced more single-strand breaks (tail length 19.88) than AuCit-NPs (9.59).

Complementary quantitative measures further support DNA damage patterns. Under alkaline conditions, AuCh-NPs consistently showed percentages of DNA in tail (0.49) similar to AuCit-NPs, while in neutral electrophoresis, this pattern reversed, with AuCh-NPs demonstrating higher values (0.50). Furthermore, tail moment measurements revealed that AuCh-NPs (1.01) are higher than those for AuCit-NPs (0.59) in alkaline conditions, and in neutral conditions, AuCh-NPs (1.01) are higher than those for AuCit-NPs (0.86).

These findings collectively demonstrate that AuCh-NPs primarily induce DNA fragmentation patterns detected predominantly in neutral comet assays (high tail moment, low %DNA in alkaline assays), characteristic of large-scale apoptotic DNA cleavage (50–300 kbp fragments). In contrast, AuCit-NPs generate widespread damage detectable in alkaline and neutral assays, suggesting non-specific double-strand breaks associated with necrosis or genotoxic stress. The differential effects between nanoparticle treatments became more pronounced following LED irradiation, with neutral comet assays proving particularly effective at distinguishing the apoptotic DNA fragmentation patterns induced by AuCh-NPs. The distinct damage profiles suggest fundamentally different mechanisms of DNA interaction between the two nanoparticle types.

## Discussion

Breast cancer patients undergoing conventional treatments often experience systemic toxicity due to the non-selective targeting of both malignant and healthy cells. In contrast, due to its demonstrated anticancer properties, folk medicine has long utilized Chaga mushroom (Inonotus obliquus) as a dietary supplement for cancer patients.

The successful synthesis of AuCh-NPs was confirmed by a characteristic peak closely resembling that of AuCit-NPs, as Chaga mushroom extract alone does not exhibit such a peak. The shift in the AuCit-NPs peak upon AuCh-NP formation can be attributed to differences in particle size, hydrodynamic behavior, and aggregation tendencies in aqueous solution. The synthesis of AuCh-NPs involved the uniform binding of active chemical groups, facilitated by reducing agents in AuCit-NPs, which converted gold ions into metallic gold, confirming successful nanoparticle formation.

The resulting AuCh-NPs exhibited nanorod morphology with a crystalline surface, enhancing their stability, cellular permeability, and retention efficacy. Notably, AuCh-NPs displayed superior cellular uptake in breast cancer cells via endocytosis compared to AuCit-NPs, amplifying their anticancer potential [24].

Our research further demonstrated that exposure to 530 nm LED light (total irradiation: 325.8 J cm^−2^) significantly enhanced the anticancer efficacy of AuCh-NPs, consistent with the findings of Sanità et al. [3]. A combination of low-dose AuCh-NPs and LED irradiation produced a pronounced anticancer effect [31], leveraging the tumor-targeting capabilities of nanomaterials while circumventing the limitations of conventional therapies, such as nonspecific biodistribution and high dosage requirements [16].

Hydrodynamic size distribution analysis revealed that AuCh-NPs had a larger and broader distribution than AuCit-NPs, suggesting a higher surface charge in citrate-stabilized NPs. This property likely reduced nanoparticle aggregation, contributing to the enhanced anticancer performance of mycosynthesized AuCh-NPs [24]. Our study highlights that AuCh-NPs uniquely combine the therapeutic properties of Chaga extract with the advantages of gold nanoparticles, exhibiting a smaller nanoscale and superior anticancer activity compared to prior chaga-derived nanoparticles [22].

In MCF-7 breast cancer cells, AuCh-NPs combined with LED-mediated photothermal therapy (PTT) demonstrated potent anticancer effects, as evidenced by the lowest IC_50_ value and a significant increase in cell cycle arrest relative to previous studies [15, 35]. Additionally, this treatment activated autophagy pathways, suppressing cell migration [13].

Mechanistically, our approach markedly increased pro-apoptotic BAX expression while decreasing anti-apoptotic factors, correlating with elevated double-strand DNA damage and apoptosis compared to other treatment groups. Our results demonstrated that cancer cells treated with AuCh-NPs and subjected to LED irradiation exhibit a pronounced apoptotic mechanism, corroborated by prior work [31]. MCF-7 cell lines that were incubated with AuCh-NPs and subjected to LED-mediated photothermal therapy showed a concentration-dependent suppression of cell cycle progression due to the inhibition of proliferation, which corroborates an increase in the apoptosis-induced killing rate, similar to findings in prior work [9].

Remarkably, the combined regimen of Chaga extract, AuCit-NPs, and LED-PPT at minimal dosages outperformed alternative therapies in anticancer efficacy [10]. Moreover, AuCh-NP-mediated PTT required significantly lower doses and shorter treatment durations (30 min) compared to studies like Zhao et al., where inositol-based treatments necessitated higher concentrations to induce cell cycle arrest.

Gene expression analysis revealed that AuCh-NP PTT significantly upregulated BAX and downregulated BCL2 in MCF-7 cells, triggering cytochrome C release and apoptosis initiation. These findings align with Mfouo-Tynga et al., who reported mitochondrial membrane disruption and cytochrome C release in nanoparticle-treated breast cancer cells.

Finally, alkaline and neutral comet assays confirmed extensive DNA damage in AuCh-NP PTT-treated cells, indicated by elongated tail lengths, higher DNA percentages, and increased tail moments compared to AuCit-NP PTT. These results parallel Shukurov et al. [7], where ZnPC-based photothermal therapy similarly induced apoptotic DNA fragmentation.

## Conclusion

Our study demonstrates that AuCh-NPs, combined with LED-mediated photothermal therapy (530 nm, 325.8 J cm^−2^), exhibit a potent anticancer effect in MCF-7 breast cancer cells by activating apoptotic pathways. These findings highlight the potential of integrating mycosynthesized AuCh-NPs into PTT as a promising, low-dose, and tumor-selective treatment strategy. Future research should explore in vivo efficacy and long-term biocompatibility to advance this approach toward clinical applications.

## Data Availability

No datasets were generated or analysed during the current study.
